# PhcA and PhcR Regulate Ralsolamycin Biosynthesis Oppositely in *Ralstonia solanacearum*

**DOI:** 10.3389/fpls.2022.903310

**Published:** 2022-05-27

**Authors:** Peng Li, Xiulan Cao, Liwen Zhang, Mingfa Lv, Lian-Hui Zhang

**Affiliations:** ^1^Ministry of Education Key Laboratory for Ecology of Tropical Islands, Hainan Provincial Key Laboratory for Tropical Plant and Animal Ecology, College of Life Sciences, Hainan Normal University, Haikou, China; ^2^Biotechnology Research Institute, The Chinese Academy of Agricultural Sciences, Beijing, China; ^3^Guangdong Province Key Laboratory of Microbial Signals and Disease Control, Integrative Microbiology Research Centre, South China Agricultural University, Guangzhou, China

**Keywords:** *Ralstonia solanacearum*, ralsolamycin, PhcA, PhcR, regulatory mechanism

## Abstract

Ralsolamycin, one of secondary metabolites in *Ralstonia solanacearum*, is known to be involved in crosstalk between *R. solanacearum* and fungi. Ralsolamycin formation is catalyzed by two-hybrid synthetases of RmyA (non-ribosomal peptide synthetase) and RmyB (polyketide synthase). A methyltransferase PhcB catalyzes formation of 3-OH MAME or 3-OH PAME, signals for the quorum sensing (QS) in *R. solanacearum*, while PhcB positively modulates ralsolamycin biosynthesis. A two-component system of PhcS and PhcR can response these QS signals and activate *phcA* expression. Here, we experimentally demonstrated that deletion of *phcA* (Δ*phcA*) substantially impaired the ralsolamycin production and expression of *rmyA* and *rmyB* in *R. solanacearum* strain EP1, and failed to induce chlamydospore formation of plant fungal pathogen *Fusarium oxysporum* f. *cubense* (stran FOC4). However, deletion of *phcR* significantly increased ralsolamycin production and expression of *rmyA* and *rmyB*, and *phcR* mutants exhibited enhanced ability to induce chlamydospore formation of FOC4. Results of the electrophoretic mobility shift assay suggested that both PhcA and PhcR bind to promoter of *rmy* operon. Taken together, these results demonstrated that both PhcA and PhcR bind to promoter of *rmy* operon, but regulate ralsolamycin biosynthesis in an opposite way. It could extend our knowledge on the sophisticated regulatory networks of ralsolamycin biosynthesis in *R. solanacearum*.

## Introduction

*Ralstonia solanacearum* is a causal agent of bacterial wilt disease on extremely broad range of plant species, which includes *R. solanacearum* and closely related species of *R. syzygii*, *R. picketti*, and banana blood disease (BDB) bacterium (Hayward, 1991). In addition, the *R. solanacearum* is also well known for extremely wide geographic distributions and capability to live and compete for versatile and diverse habitats (Salanoubat et al., 2002; Alvarez et al., 2010).

Interaction between *R. solanacearum* and other microorganisms in virulence or environmental competition has recently attracted much attention, and secondary metabolites (SMs) plays important roles on inter-kingdom signaling communication or provides fitness advantages in dynamic polymicrobial ecosystems (Baldeweg et al., 2017). The ralfuranone family contribute to virulence of *R. solanacearum* strain OE1-1(Kai et al., 2014; Senuma et al., 2020), while the staphyloferrin B is a siderophore associated with iron scavenge in strain AW1, which has a role in the competitiveness of phytopathogen outside its host plants (Bhatt and Denny, 2004). The yersiniabactin-like siderophore micacocidin was identified as an anti-mycoplasma agent (Kreutzer et al., 2011). Recently, ralsolamycin (synonym ralstonin A) is catalyzed by two-hybrid synthetases of RmyA (non-ribosomal peptide synthetase) and RmyB (polyketide synthase) that works as an inter-kingdom signal to communicate with fungal organisms and induces chlamydospores formation of interacted fungi. (Spraker et al., 2016; Baldeweg et al., 2017; Murai et al., 2017). As a response to ralsolamycin induction, fungi enhance bikaverin biosynthesis to antagonize *R. solanacearum* (Spraker et al., 2018). These findings indicate that *R. solanacearum* is capable of a producer of diverse SMs, which has important implications for the persistence of this phytopathogen.

It has been well known that the bacterial populations can emit and detect small diffusing compounds, whose concentration is key for coordinated behaviors. The quorum sensing (QS) is such a process that allows bacterial population to regulate specific genes at the quorum cell density (Fuqua et al., 1994). In *R. solanacearum*, either methyl 3-hydroxypalmitate (3-OH PAME) and methyl 3-hydroxymyristate (3-OH MAME), signals for phc-QS system, which are encoded by a methyltransferase PhcB (Flavier et al., 1997; Kai et al., 2015). The phc-QS signals are sensed through kinase PhcS, a sensor histidine of a two-component system (TCS), and transferred to PhcA, a TCS transcriptional regulator, which in turn activates transcriptional ability of PhcR, a LysR family transcriptional regulator to globally control many phc-QS-dependent phenotypes including virulence (Genin and Denny, 2012). Genes of *phcB*, *phcS*, and *phcR* are located together that possibly form an operon to control the phc-QS system in *R. solanacearum*, which is therefore called the phc-QS system (Clough et al., 1997).

We previously demonstrated that deletion of *phcB* drastically decreased expression of *rmyA* and *rmyB* and impaired chlamydospores formation in a *Fusarium oxysporum* f. *cubense* (FOC4), soil-borne phytopathogens that exhibits attenuated antifungal activity against *Sporisorium scitamineum* (Li et al., 2017). In addition, expression of *rmyA* and *rmyB* is downregulated with *phcA* deletion (Perrier et al., 2018). Whereas it remains to be elucidated whether *phcA* mutants affects ralsolamycin production in *R. solanacearum* and chlamydospores formation in FOC4. The *phcB*, *phcS*, and *phcR* form an operon and PhcR modulates transcriptional activity of PhcA. We therefore focused on PhcA and PhcR to elucidate whether they modulate ralsolamycin biosynthesis in *R. solanacearum* and chlamydospores formation in FOC4.

In this study, we constructed the deletion mutants of *phcA* (named Δ*phcA*) and *phcR* (named Δ*phcR*), respectively, using EP1 as a parental strain (Li et al., 2016). The expression level of *rmy* genes was compared by quantitative real-time polymerase chain reaction (qRT-PCR), and the ralsolamycin production was analyzed by using matrix-assisted laser desorption ionization time-of-flight spectrometry (MALDI-TOF). Our results demonstrated that both PhcA and PhcR were involved in the modulation of ralsolamycin biosynthesis directly but with opposite modulation patterns. The findings from this study outlined the regulatory networks that govern ralsolamycin biosynthesis, suggesting that *R. solanacearum* has evolved delicate regulatory mechanisms to respond and adapt to changing environmental conditions.

## Materials and Methods

### Bacterial and Fungal Strains, Plasmids, and Culture Conditions

Plasmids, *R. solanacearum*, *Escherichia coli*, and *F. oxysporum* f. *cubense* used in this study were listed in Table 1. *R. solanacearum* phylotype I strain EP1 causes virulence on tomato plants but elicits hypersensitive response (HR) on tobacco plants, and it was subjected as parent strain for mutant generation in this study (Li et al., 2016). *R. solanacearum* strains were grown at 28°C in casamino acid–peptone–glucose (CPG) plate (Hendrick and Sequeira, 1984) or minimal medium (MM) plate. *E. coli* DH5*α* was grown at 37°C in LB medium and used for plasmid construction. *F. oxysporum* f. *cubense* FOC4 was grown at 30°C in PDA medium. Antibiotics were supplemented when necessary at following concentrations (μg/L): ampicillin, Amp (100); kanamycin, Km (50); and rifampicin, Rif (30).

**Table 1:** List of the bacterial and fungal strains and plasmids used in this study.

Strain/Plasmid	Relevant characteristics	Source
***R. solanacearum***		
EP1	Wild type, Rif^r^	Li et al. (2016); CFBP8480
Δ*phcA*	*phcA* deletion mutant of EP1 (Rif^r^)	This study
Δ*phcA*(*phcA*)	Δ*phcA* + *phcA* (Km^r^, Rif^r^)	This study
Δ*phcR* Δ*phcR*(*phcR*)	*phcR* deletion mutant of EP1 (Rif^r^) Δ*phcR* + *phcR* (Km^r^, Rif^r^)	This study This study
**Fungus**		
FOC4	*F. oxysporum* f. *cubense*	Li et al. (2014)
**Plasmids**		
pK18mobsacB	Km^r^, suicide, and narrow-broad-host vector	Schäfer et al. (1994)
pBBR1MCS2	Km^r^, broad-host-range cloning vector	Kovach et al. (1995)
pRK2013	Km^r^	Ditta et al. (1980)
pET-32a-*phcA*	pET-32a carries the *phcA* coding region, Amp^r^	This study
pET-32a-*phcR*	pET32a carries the *phcR* coding region, Amp^r^	This study

### Mutants Generation With In-frame Deletion of Genes and Complementation Assay

In the present study, mutants were generated with the pK18mobsacB based homologue recombination, by which target genes were in-frame deleted as described previously (Li et al., 2017). In brief, coding sequence of target genes was removed by the joint PCR, which conjugated both ends of flanking DNA fragments. The PCR amplified DNA fragments, in which target genes were absent, were finally sub-cloned into suicide plasmid pK18mobsacB. All primers used in this study were listed in Supplementary Table S1. After validating sequences, recombinant plasmids helped by the pRK2013 were introduced into EP1 by the tri-parental mating on CPG plates, followed by enrichment of integration mutants of strain EP1 on MM plates supplemented with Km and Rif as described previously (Ditta et al., 1980). As results, the *phcA* and *phcR* mutants were generated after confirmed by colony PCR and DNA sequencing.

The genetic complementation was performed with the expression vector pBBR1MCS2-based complementation assay as described previously (Kovach et al., 1995). In brief, DNA fragments containing putative promoter and coding sequence of *phcA* and *phcR* were PCR amplified and finally sub-cloned into pBBR1MCS2, respectively. Genes of *phcB*, *phcS*, and *phcR* from an operon and the putative promoter of this operon (*phcB* upstream region about 235 bp) were PCR conjugated with coding sequence of *phcR*. After validating sequences, recombinant plasmids were introduced into corresponding mutants with the above tri-parental mating system, and complementary strains were confirmed by colony PCR and DNA sequencing.

### RNA Preparation and qRT-PCR Analysis

*R. solanacearum* strains were grown to an OD_600_ of about 1.0 and total RNA were extracted by using a RNeasy Mini Kit (QIAGEN, Hilden, Germany). Contaminated genomic DNA was digested with DNaseI (Takara, Dalian, China) and confirmed by PCR using the primer pair for 16S rDNA. The cDNA was synthesized using the FastQuant cDNA first chain synthesis Kit (TIANGEN BIOTECH CO. LTD, Beijing, China) according to the manufacturer’s instructions. qRT-PCR was performed with Super Real PreMix Color SYBR Green (TIANGEN BIOTECH CO. LTD, Beijing, China) on Light Cycler480II (Applied Biosystems by Roche, Germany). The absolute value of –ΔΔCt = −(ΔCt1–ΔCt2) were calculated as described in the 2^-ΔΔCt^ method (Livak and Schmittgen, 2001). Each assay was repeated from RNA isolation for three independent experiments with at least three replications per trial. All the Ct data of *rmy* genes, *phcA* and *phcR* in EP1 and related mutants have been listed in Supplementary Tables S2-S4.

### MALDI-TOF and LC-MS Analysis

In this study, chemical compounds in bacterial extracts were detected and quantified with matrix-assisted laser desorption ionization time-of-flight spectrometry (MALDI-TOF). Ralsolamycin could be detected at the peak of *m*/*z* 1291.7142 with the MALDI-TOF and its content could be quantified by calculating the peak areas. For the MALDI-TOF analysis, a tiny number of colonies were scratched off from agar plates and transferred to a MALDI MSP 384 anchor-chip plate, followed by application of 2 μl universal matrix (1:1 mixture of 2, 5-dihydroxybenzoic acid and α-cyano-4-hydroxy-cinnamic acid). The plate was dried at 37°C for 1 h and analyzed using a Bruker Autoflex MALDI-TOF mass spectrometer (Bruker Daltonics, Billerica, MA, United States) in positive reflectron mode, with a mass range of 500–3,500 Da. Each assay was repeated for three independent experiments with four replications per trial. The natural log of data obtained was analyzed using FlexAnalysis 3.0 software (Bruker Daltonics, Billerica, MA, United States).

### Electrophoretic Mobility Shift Assay

Binding of proteins onto DNA probes was determined with the electrophoretic mobility shift assay (EMSA) as described previously (Lv et al., 2018). PhcA and PhcR proteins were purified from the pET-32a based protein expression system in *E. coli* BL21 (DE3) and subjected for the binding reaction with biotinylated DNA probes using the LightShift Chemiluminescent EMSA Kit following the manufacturer’s protocol (Thermo, United States). Promoter regions of the *rmyA* operon (*rmyA* upstream region of ATG site about 375 bp) and *impH* gene (424 bp of its coding sequence) were PCR amplified and labeled by biotin using the Biotin 3′ End DNA Labeling Kit (Thermo, United States). Note that promoter DNA of *impH* gene, which encodes one of the type VI secretion proteins in *R. solanacearum*, was selected as a system control. The gels were stained by SYBR GOLD dye and subjected to screening on a phosphor screen (LAS600, GE Healthcare).

### Interaction Between *Ralstonia solanacearum* and *Fusarium oxysporum* f. *cubense* FOC4

*R. solanacearum* strains and *F. oxysporum* f. *cubense* FOC4 were dropped onto same PDA agar plate and incubated at 28°C for about eight days. When two kinds of colonies interweaved each other, the FOC4 mycelia surrounded the interaction zones at about 1 cm were harvested and subjected for chlamydospore counting under microscope (Li et al., 2017).

### Statistical Analysis

Each assay was repeated for three independent experiments with at least three replications per trial. Mean values of all experiments were averaged with SD (error bars) and statistical significance was assessed using a paired two-tailed Student’s *t*-test by using the GraphPad Prism 6.0 software (GraphPad, La Jolla, CA). ^*^p ≤ 0.05; ^**^0.001 < *p* < 0.01; ^***^ *p* ≤ 0.001.

## Results

### PhcA Positively Regulates Ralsolamycin Production and Expression of *rmy* Genes

**Figure 1:**
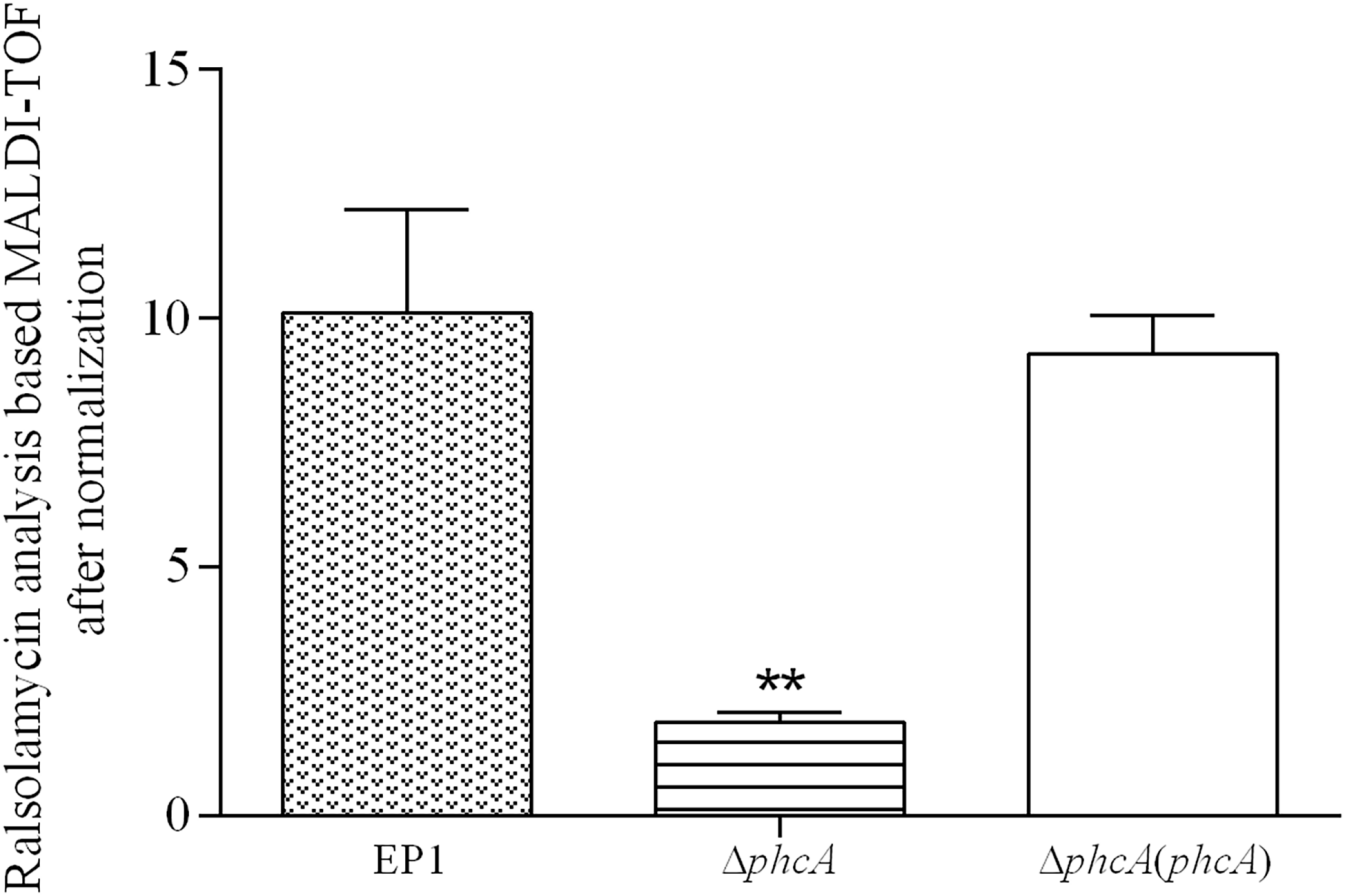
MALDI-TOF analysis of ralsolamycin (*m*/*z* 1291.7142) production in wide type strain EP1, *phcA* deletion mutant (Δ*phcA*), and complementary strain Δ*phcA*(*phcA*). Briefly, a tiny number of colonies were scratched off from agar plates and transferred to a MALDI MSP 384 anchor-chip plate, followed by application of 2 μl universal matrix. The plate was dried at 37°C for 1 h and analyzed using a Bruker Autoflex MALDI-TOF mass spectrometer in positive reflectron mode, with a mass range of 500–3,500 Da. Each assay was repeated for three independent experiments with four replications per trial. The natural log of data obtained were analyzed using FlexAnalysis 3.0 software. Mean values of all experiments were averaged with SD (error bars) and statistical significance was assessed using a paired two-tailed Student’s *t*-test by using the GraphPad Prism 6.0 software. ^**^ means 0.001 < *p* ≤ 0.05.

**Figure 2:**
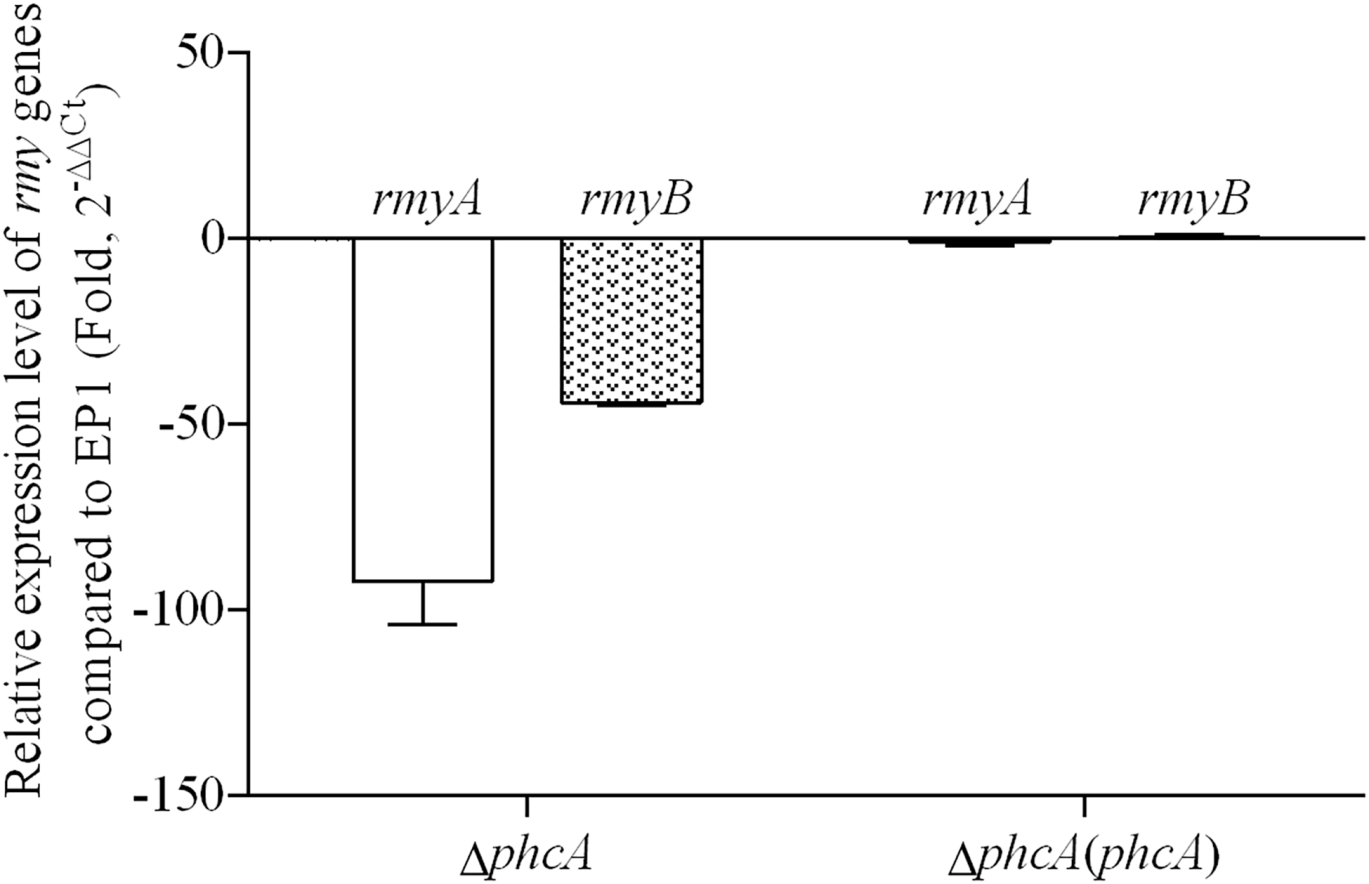
qRT-PCR analysis of *rmyA* and *rmyB* in wide type strain EP1, *phcA* deletion mutant (Δ*phcA*), and complementary strain Δ*phcA*(*phcA*) (OD_600_ = 1.0). The results were calculated as 2^–ΔΔCt^, –ΔΔCt = −(ΔCt_mutant_ –ΔCt_wt_). Three biological repeats (independent cultures) and three technical repeats were done to calculate and compare the values. Mean values of all experiments were averaged with SD (error bars) by using the GraphPad Prism 6.0 software.

PhcA is a QS-dependent transcriptional regulator that is activated through the phc-QS signals and in turn regulates many QS-dependent phenotypes, including ralfuranones, exopolysaccharide (EPS), and virulence (Brumbley et al., 1993; Kai et al., 2014). We previously reported that ralsolamycin biosynthesis was modulated by the phc-QS system in *R. solanacearum* EP1 (Li et al., 2017). We therefore assessed whether PhcA regulates ralsolamycin biosynthesis in EP1. Ralsolamycin production was quantified with the MADLI-TOF analysis at the peak of *m*/*z* 1,291. Deletion of *phcA* substantially decreased ralsolamycin production, which was approximately 20% of that in EP1, and complementation of *phcA* fully restored the decreased ralsolamycin production (Figure 1). The *rmy* operon of *rmyA* and *rmyB* genes are responsible for ralsolamycin biosynthesis in *R. solanacearum* (Spraker et al., 2016). We further assessed whether *phcA* deletion decreased expression levels of *rmyA* and *rmyB* genes. Total RNA was isolated from the wild-type strain EP1, *phcA* mutants, and its complementary strains, and mRNA levels of *rmyA* and *rmyB* were quantified with the qRT-PCR. Deletion of *phcA* substantially decreased mRNA levels of *rmyA* and *rmyB*, and complementary *phcA* fully restored the decreased transcriptional levels (Figure 2). These results confirmed that PhcA positively regulates expression of *rmy* genes and in turn modulates ralsolamycin biosynthesis.

### PhcR Negatively Regulates Ralsolamycin Production and Expression of *rmy* Genes

**Figure 3:**
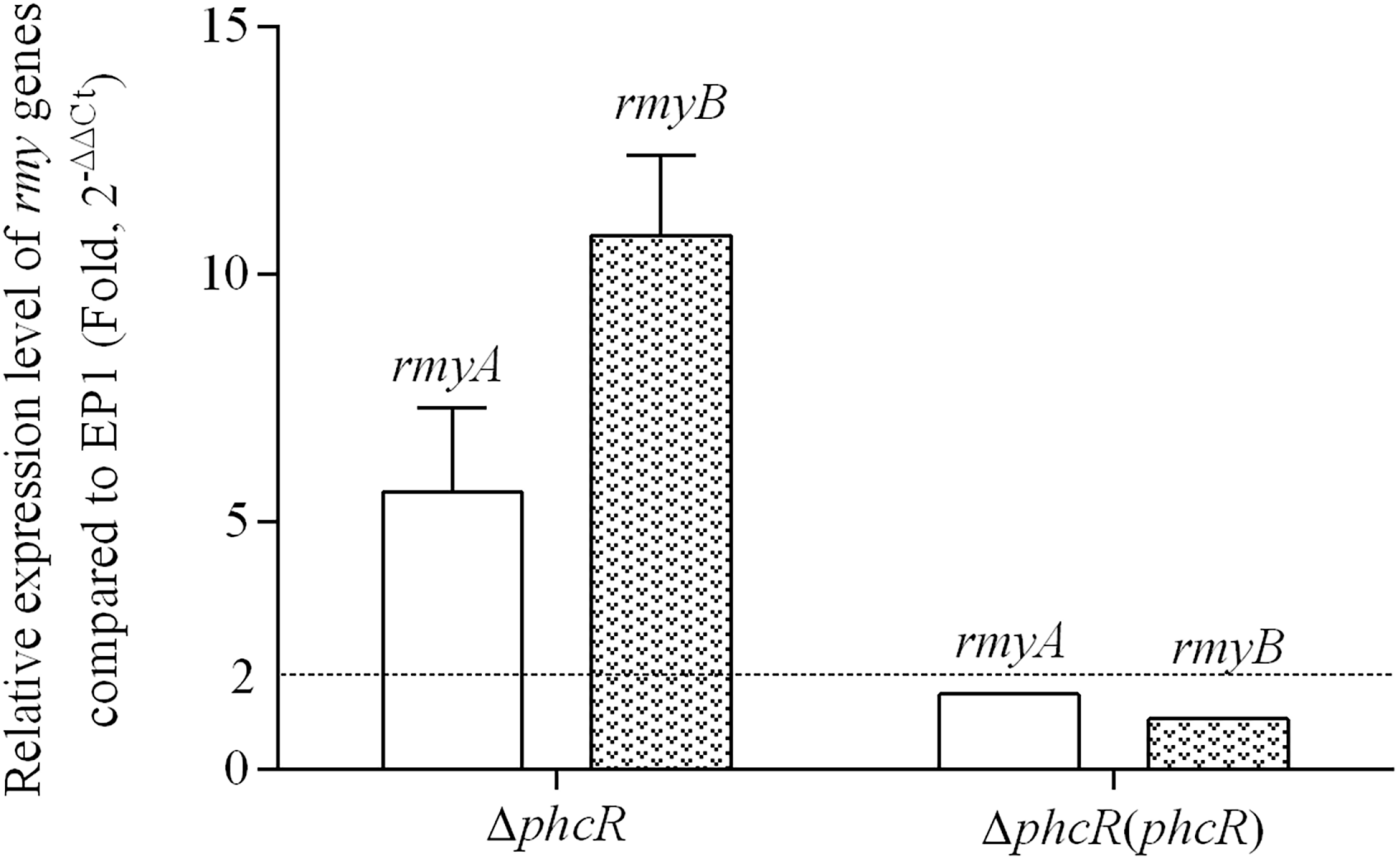
qRT-PCR analysis of *rmyA* and *rmyB* in wide type strain EP1, *phcR* deletion mutant (Δ*phcR*) and complementary strain Δ*phcR* (*phcR;* OD_600_ = 1.0). The results were calculated as 2^–ΔΔCt^, –ΔΔCt = −(ΔCt_mutant_ –ΔCt_wt_). Three biological repeats (independent cultures) and three technical repeats were done to calculate and compare the values, mean values of all experiments were averaged with SD (error bars) by using the GraphPad Prism 6.0 software.

**Figure 4:**
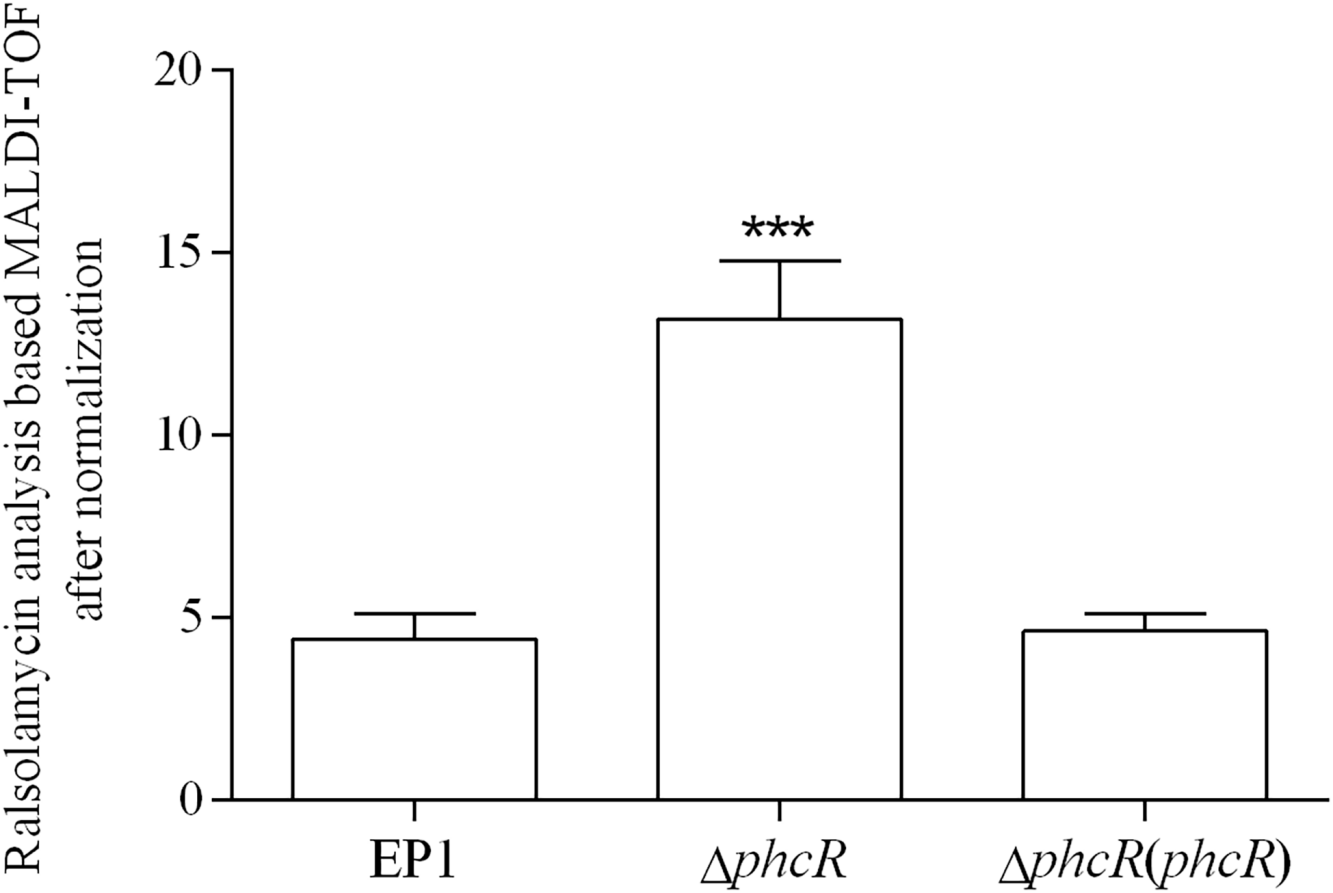
matrix-assisted laser desorption ionization time-of-flight spectrometry analysis of ralsolamycin production in wide type strain EP1, *phcR* deletion mutant (Δ*phcR*) and complementary strain Δ*phcR* (*phcR*). Presence of the peak in the bacterial extract showing the accurate mass of ralsolamycin (*m*/*z* 1291.7142) was identified, and the corresponding peak areas of wild type and mutants were compared and calculated. Each assay was repeated for three independent experiments with four replications per trial. The natural log data obtained were analyzed using FlexAnalysis 3.0 software. Mean values of all experiments were averaged with SD (error bars) and statistical significance was assessed using a paired two-tailed Student’s *t*-test by using the GraphPad Prism 6.0 software. ^***^ means *p* ≤ 0.001.

The *phc* operon consists of *phcB*, *phcS*, and *phcR*, which encode the phc-QS system. The *phcB* is responsible for biosynthesis of phc-QS signals 3-OH PAME or 3-OH MAME (Flavier et al., 1997; Kai et al., 2015). The sensor histidine kinase PhcS can sense these QS signals and activate the phc-QS system *via* PhcR (Clough et al., 1997). We previously demonstrated that PhcB is involved in positive regulation on *rmy* expression and ralsolamycin production (Li et al., 2017). We thus assessed whether PhcR regulates ralsolamycin biosynthesis in EP1. Differently from that in *phcA* mutants, deletion of *phcR* significantly enhanced mRNA levels of *rmyA* and *rmyB*, and complementary *phcR* substantially decreased enhanced mRNA levels of *rmyA* and *rmyB* in *phcA* mutants (Figure 3), indicating that PhcR plays a negative role on *rmy* expression. Ralsolamycin production was quantified in *phcR* mutants with the MADLI-TOF analysis, and *phcR* deletion significantly increased the ralsolamycin production, which was about three folders of that in the wild-type strain (Figure 4). Complementary *phcA* substantially decreased the increased ralsolamycin production to levels of the wild-type strain (Figure 4). These results confirmed that PhcR negatively regulates expression of *rmy* genes and in turn modulates ralsolamycin biosynthesis.

### PhcA and PhcR Affect Chlamydospore Formation of FOC4 in an Opposite Way

**Figure 5:**
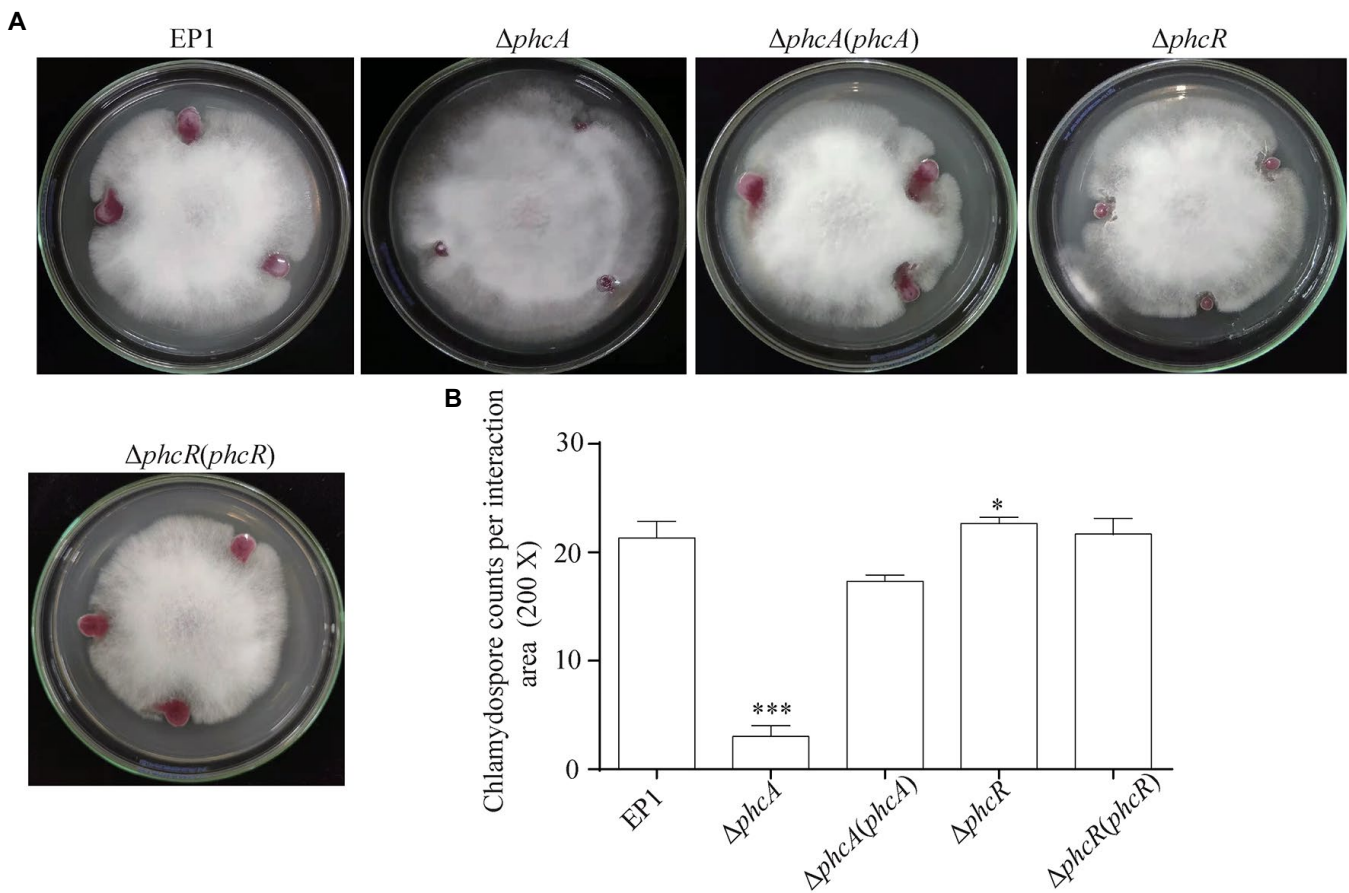
Coculture experiment between *R. solanacearum* and FOC4 **(A)** and the chlamydospores counts (200 magnification times) in their interaction zones **(B)**. Δ*phcA* and Δ*phcR* refers to deletion of *phcA* and *phcR* from wide type strain EP1, respectively; Δ*phcA* (*phcA*) and Δ*phcR* (*phcR*) refers to the complementary strain of Δ*phcA* and Δ*phcR*, respectively. Each assay was repeated for three independent experiments with at least three replications per trial. Mean values of all experiments were averaged with SD (error bars) and statistical significance was assessed using a paired two-tailed Student’s *t*-test by using the GraphPad Prism 6.0 software. ^*^ means *p* < 0.05, ^***^ means *p* < 0.001.

The ralsolamycin produced by *R. solanacearum* plays important roles on interaction between *R. solanacearum* and fungi that can induce chlamydospore formation in fungi (Spraker et al., 2016). PhcA and PhcR play opposite regulatory roles on ralsolamycin production. We further assessed whether PhcA and PhcR affect interaction between *R. solanacearum* and *F. oxysporum* f. *cubense* FOC4. *R. solanacearum* and FOC4 were dropped onto same PDA plate, and then development of mycelia and chlamydospore production of FOC4 was evaluated (Li et al., 2017). The wild-type strain EP1 and *phcR* mutants could form clear inhibition haloes against FOC4 mycelia at four to 10 days post-inoculation (dpi), while *phcA* mutants failed to form inhibition haloes even till to 10 dpi and FOC4 mycelia could cover colonies of *phcA* mutants (Figure 5A). Complementary *phcA* or *phcR* restored corresponding changed phenotypes in each mutant, respectively (Figure 5A). The chlamydospores formation of FOC4 in the interaction zones were counted under microscope at 10 dpi. Chlamydospore quantities were substantially impaired when co-cultured with *phcA* mutants, which was approximately 15% of the control with EP1 and complementation of *phcA* substantially restored the impaired chlamydospore formation (Figure 5B). On the contrary, chlamydospore formation was slightly increased when co-cultured with *phcR* mutants (*p* = 0.035168; Figure 5B). These results confirmed that PhcA and PhcR affect chlamydospore formation of FOC4 in an opposite way.

### Both PhcA and PhcR Can Bind Directly to Promoter DNA of *rmy* Operon Genes

**Figure 6:**
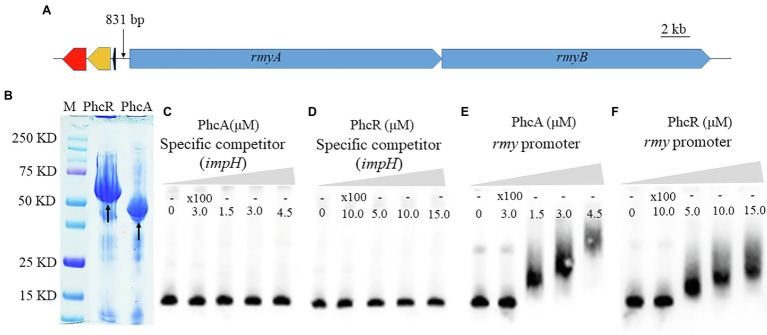
Schematic presentation of *rmyA* and *rmyB* operons **(A)**, proteins (PhcA and PhcR) expression **(B)** and promoter binding detection by electrophoretic mobility shift assay **(E,F)**. 30 fmol of labeled DNA fragments corresponding to the promoter region of *rmyA* gene was incubation with 100 nm and 200 nm PhcA/R, respectively, using 100-fold unlabeled corresponding DNA fragments as the specific competitor. The coding sequence of *impH* (424 bp), which encodes a type VI secretion protein and is not bound by PhcA and PhcR, was used as a negative control in the EMSA experiment **(C,D)**. The positions of free DNA and of protein–DNA complexes were shown.

Given the fact that PhcA and PhcR regulated expression of *rmyA* an *rmyB* genes, we evaluated whether these two transcriptional regulators could bind directly to promoter DNA of *rmy* operon genes with the EMSA. Genes *rmyA* and *rmyB* are located together and form a *rmy* operon to work together for ralsolamycin biosynthesis. The spacer between *rmyA* and its prior gene is 831 bp, a sequence of 375-bp located before the ATG site of *rmyA* was amplified as the promoter DNA, which empirically harbors the native promote and indeed worked as promote to fulfill above complementation assays. In addition, 424 bp of *impH* coding region, one of the T6SS genes, was PCR amplified as a system control to assess whether PhcA and PhcR proteins could bind to any the promoter DNA. PhcA and PhcR proteins failed to bind to the coding sequence of *impH*, but bound to the promoter DNA of *rmy* operon (Figure 6), indicating that both PhcA and PhcR can bind directly to promoter DNA of *rmy* operon genes.

### PhcA and PhcR Was Independent of Either Transcriptional Expression

**Figure 7:**
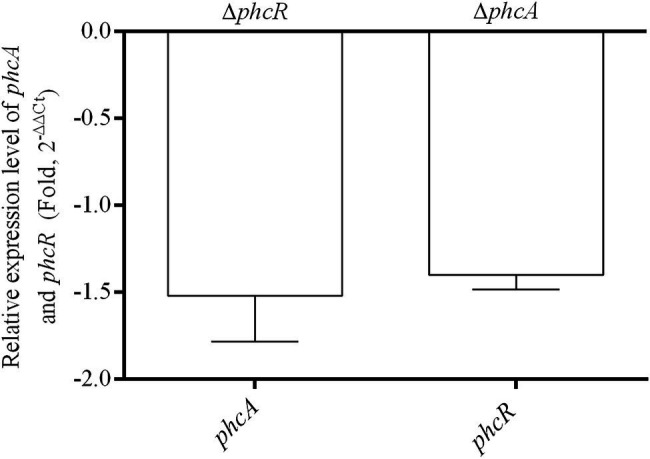
qRT-PCR analysis of *phcA* and *phcR* gene expression in Δ*phcR* and Δ*phcA* mutant of strain EP1, respectively (OD_600_ = 1.0). The Δ*phcA* and Δ*phcR* refers to deletion of *phcA* and *phcR* from wide type strain EP1, respectively. The results were calculated as 2^–ΔΔCt^, –ΔΔCt = −(ΔCt_mutant_ –ΔCt_wt_). Three biological repeats (independent cultures) and three technical repeats were done to calculate and compare the values, mean values of all experiments were averaged with SD (error bars) by using the GraphPad Prism 6.0 software.

We further evaluated whether PhcA or PhcR could regulate transcriptional expression of the other gene with the qRT-PCR. Total RNA was isolated and mRNA levels of *phcA* and *phcR* were quantified with the qRT-PCR. Deletion of *phcA* did not alter mRNA levels of *phcR*, and vice versa (Figure 7), indicating that PhcA and PhcR were independent of transcriptional expression of the other gene.

## Discussion

*R. solanacearum* can survive in various environmental conditions, partially due to its ability to produce a variety of SMs which aids to its ability in competition with other living organisms (Delaspre et al., 2007; Schneider et al., 2009; Kreutzer et al., 2011). Among them, ralsolamycin plays an important role in interspecific interaction and cross-kingdom communications, which has profound impacts on the ability of *R. solanacearum* to colonize and survive in complex biotic environments. Our previous study reported that ralsolamycin biosynthesis was decreased distinctly when the phc-QS signal coding gene *phcB* was deleted, it is not yet clear what other regulators are involved in modulation of ralsolamycin biosynthesis. In this study, we provided multiple lines of evidences to demonstrated that both PhcA and PhcR can regulate the expression of *rmy* genes and affect the ralsolamycin biosynthesis, as well as the interaction between *R. solanacearum* and the fungal pathogen FOC4. Interestingly, our results confirmed that PhcA is the positive regulator modulating the ralsolamycin biosynthesis, while PhcR is the negative regulator. Understanding the behavior and mechanisms involved in the interaction among environmental microbe that share ecological niches is fundamental in the study of microbial population adaptation and evolution (Martin et al., 2017). Thus, it is important to decipher how the ralsolamycin biosynthesis is regulated. In this work, we provide new insights into the positive and negative regulation mechanisms that conducted by PhcA and PhcR, respectively.

Extensive experiments have confirmed that PhcA is a global transcriptional regulator (Brumbley et al., 1993), which works as a molecular switch can directly or indirectly regulate the production of numerous virulence determinants, such as EPS, cell wall-degrading enzymes, chemotaxis system, and secretion systems (Denny and Ryel, 1991; Arlat et al., 1992; Huang et al., 1995; Yao and Allen, 2006; Genin and Denny, 2012). Consequently, benefited from those virulence determinants, *R. solanacearum* owns excellent ability to infect numerous plant hosts. In addition to the role in regulation of virulence, PhcA also regulates traits which help *R. solanacearum* to survive in nutrient-poor environments and to grow rapidly during early pathogenesis (Khokhani et al., 2017). Consistent with the above findings, our results showed that the *rmyA* and *rmyB* genes, which encode production of ralsolamycin, were positively regulated by PhcA. The findings that ralsolamycin production was significantly decreased in the PhcA null mutant validates that PhcA involves in the regulation of ralsolamycin biosynthesis (Figure 1), which was further confirmed by qRT-PCR analysis (Figure 2). According to the results of ralsolamycin production, deletion of *phcA* almost abolished the ralsolamycin production, it is reasonable to think that PhcA is the key regulator involved in modulation of ralsolamycin biosynthesis. In the absence of *phcA* shows a dry clone and loss the ability that inducing chlamydospore formation of fungi, as well as the competitive ability with fungi (Figure 5). Thus, our results identify a new regulator associated with the modulation of ralsolamycin production and the bacterium–fungus interaction and add a new list to the regulatory spectrum of the master regulator PhcA in *R. solanacearum*.

Our previous results showed that ralsolamycin production is under the regulation of the phc-QS system. Deletion of the QS signal encoding gene *phcB* caused a significant reduction in ralsolamycin production (Li et al., 2017). Toward this end, we explored the role of PhcR, which is believed to response to the phc-QS signal, in the modulation of ralsolamycin production. Results showed that the *rmy* gene expression and production of ralsolamycin were increased when the *phcR* was deleted (Figures 3, 4). Meanwhile, the ability to induce chlamydospore formation was also increased (Figure 5). Thus, PhcR showed an opposite regulatory pattern with PhcB and PhcA. Similar results were obtained with transcriptome analysis that PhcR is partially involved in the regulation of QS-dependent genes in *R. solanacearum* strain OE1-1, such as the genes *norB*, *lecM*, and *xpsR* were modulated by PhcR positively, and some flagellin biosynthesis-related genes and chemotaxis-related genes were negatively PhcR-dependent genes (Takemura et al., 2021). In addition, the similar regulatory patterns were also found in modulation of the biosynthesis of ralfuranone, which is a SM and virulence factor produced by *R. solanacearum* (Wackler et al., 2011), for the ralfuranone coding gene expression and biosynthesis is regulated positively by PhcA and negatively by PhcR (Takemura et al., 2021). However, it is not yet clear whether PhcA and PhcR regulate the ralfuranone gene expression in a direct way similar to what we have found for the regulation of ralsolamycin. Moreover, in the present study, we uncovered that the gene expression level of *phcA* and *phcR* were not affected when the *phcR* and *phcA* gene was deleted, respectively (Figure 7), PhcR and PhcA were thus all found to be independently involved in the control of biosynthesis of ralsolamycin. In conclusion, it is intriguing that both PhcR and PhcA bind to same promoter but display opposite regulation on the *rmy* genes expression. However, how the two regulators targeting the same promoter have opposite regulatory effects and how this regulation is carried out in a wild-type context is still needed to be studied and resolved in the further research.

Nevertheless, these findings suggest that PhcA and PhcR play key roles in modulation of SM production. A recent study showed that plant sugars d-galactose and d-glucose could activate the production of ralfuranones in *R. solanacearum* (Ishikawa et al., 2019), suggesting that both QS and cross-kingdom chemical communications play a role in regulation of the SM. Further investigation of the response of PhcA and PhcR to various signal inputs would aid to understand their detailed regulatory mechanisms.

In conclusion, this study demonstrated that the ralsolamycin production is modulated by the global regulator PhcA and the phc-QS signal response protein PhcR directly at the transcriptional level with PhcA acting as the positive regulator and PhcR being the negative regulator. The opposite roles of PhcA and PhcR suggest complex and sophisticated regulatory networks that have been evolved in *R. solanacearum* to modulate the production of ralsolamycin and other SMs, which collectively contribute to the bacterial virulence and competitive ability against other living organisms. Taken together, this work extends our understanding on the intricacies of the regulatory networks of the phc-QS system in modulation of secondary metabolism, and highlights the possibility of cross-talking between QS and other signaling mechanisms in regulation of ralsolamycin biosynthesis in *R. solanacearum*.

## Data Availability Statement

The original contributions presented in the study are included in the article/Supplementary Material, further inquiries can be directed to the corresponding authors.

## Author Contributions

PL and L-HZ designed the experiments and wrote the paper. LZ performed the MALDI-TOF analysis. XC performed the qRT-PCR analysis. ML performed the EMSA experiment. L-HZ revised the manuscript. All authors contributed to the article and approved the submitted version.

## Funding

This work was supported by Hainan Province key R&D Project (ZDYF2020080), the National Natural Science Foundation of China (nos. 31901846 and 31901843), Guangdong Forestry Science and Technology Innovation Project (2018KJCX009 and 2020KJCX009), and the specific research fund of the Innovation Platform for Academicians of Hainan Province (YSPTZX202130).

## Conflict of Interest

The authors declare that the research was conducted in the absence of any commercial or financial relationships that could be construed as a potential conflict of interest.

## Publisher’s Note

All claims expressed in this article are solely those of the authors and do not necessarily represent those of their affiliated organizations, or those of the publisher, the editors and the reviewers. Any product that may be evaluated in this article, or claim that may be made by its manufacturer, is not guaranteed or endorsed by the publisher.

## Supplementary Material

Supplementary Table S1 Primers used in this study.

Supplementary Table S2 Ct data of *rmyA* and *rmyB* genes in wide type strain EP1, *phcA* deletion mutant (*ΔphcA*) and complementary strain *ΔphcA(phcA)*, cell density is OD_600_ = 1.0.

Supplementary Table S3 Ct data of *rmyA* and *rmyB* genes in EP1, *phcR* deletion mutant (*ΔphcR*) and complementary strain *ΔphcA(phcA)*, cell density is OD_600_ = 1.0.

Supplementary Table S4 Ct data of *phcA* and *phcR* gene in EP1 and mutants (OD_600_=1.0), cell density is OD_600_ = 1.0.
