# Supplementary material for: PhcA and PhcR Regulate Ralsolamycin Biosynthesis Oppositely in Ralstonia solanacearum
Source: Front Plant Sci. 2022 May 27;13:903310. doi: 10.3389/fpls.2022.903310 (PMC9197120; doi:10.3389/fpls.2022.903310)
Supplement: Supplementary Table S1 — Primers used in this study. [file Table_1.docx]

Table 1. Primers used in this study

| Primer | Prime sequence | Note |
| --- | --- | --- |
| *phcA*-L1 | CGGGATCCTGAACCACGGCACCTACAAG | *Bam*HI |
| *phcA*-L2 | GCCGCGACTCATCCTCCTTATCGACGTTGACCATGGGACG |  |
| *phcA*-R1 | CGTCCCATGGTCAACGTCGATAAGGAGGATGAGTCGCGGC |  |
| *phcA*-R2 | CCAAGCTTTCGAACACCCGGCCAGCAG | *Hin*dIII |
| *phcA*-CF | CCAAGCTTGGATTTCCGCGGCGATGCAC | *Hin*dIII |
| *phcA*-CR | CGGGATCCGCATGCCTTGTCGCTGTACG | *Bam*HI |
| *phcR*-L1 | CGGGATCCTGTAGTCCTTCAGTGTCCAG | *Bam*HI |
| *phcR*-L2 | GGAATAGGAGCGAGCATGGAATGAACCGAGCGATACCAGG |  |
| *phcR*-R1 | CCTGGTATCGCTCGGTTCATTCCATGCTCGCTCCTATTCC |  |
| *phcR*-R2 | CCAAGCTTGCTGCACGAGATCAACAACC | *Hin*dIII |
| *phcR*-C1 | CCAAGCTTGGCCTCTCCTCCAATCATCTCG | *Hin*dIII |
| *phcR*-C2 | GTGTCGGGCGTGGCGTCCATGGTGCGAATTTGCCGGAG |  |
| *phcR*-C3 | CTCCGGCAAATTCGCACCATGGACGCCACGCCCGACAC |  |
| *phcR*-C4 | CGGGATCCTCATGGGAACGTCAGGGTCA | *Bam*HI |
| 16S-F | CTGGAATCGCTAGTAATCG | qRT-PCR |
| 16S-R | AGGCTAACTACTTCTGGTAA | qRT-PCR |
| *rmyA*-F | TCAAGGCAATACGACAAG | qRT-PCR |
| *rmyA*-R | CGTCTTCATCGGTATCTC | qRT-PCR |
| *rmyB*-F | GTTCTCGACTTCGTTGA | qRT-PCR |
| *rmyB*-R | GAAGGCACCGTATTGAT | qRT-PCR |
| phcA_L | GCGAGCACATCTTCAT | qRT-PCR |
| phcA_R | CCTTCATCAGCGAGTT | qRT-PCR |
| phcR_L | CGACCATCCTGTATGTG | qRT-PCR |
| phcR_R | TTCTTCCACGCTGTTG | qRT-PCR |
| *inpH-*F | TCGTCGGCAACTGGTATCGC | EMSA |
| *inpH -*R | CATGGCGGCAAGGGCTCCA | EMSA |
| *rmy-*F | CTCGCATTATCGAATCGGGC | EMSA |
| *rmy-*R | CATAGACGGCCTTGGTTTTAGTGG | EMSA |
| pET32a-*phcA*-F | GCCATGGCTGATATCGGATCCATGGTCAACGTCGATACCAAGC | EMSA |
| pET32a-*phcA*-R | CTCGAGTGCGGCCGCAAGCTTTCAGACGGACAGCCGCGA | EMSA |
| pET32a-phcR-F | GCCATGGCTGATATCGGATCCATGGACGCCACGCCCGAC | EMSA |
| pET32a-phcR-R | CTCGAGTGCGGCCGCAAGCTTTCATGGGAACGTCAGGGTCA | EMSA |
